# Positive Selection Drove the Adaptation of Mitochondrial Genes to the Demands of Flight and High-Altitude Environments in Grasshoppers

**DOI:** 10.3389/fgene.2018.00605

**Published:** 2018-12-05

**Authors:** Xiao-Dong Li, Guo-Fang Jiang, Li-Yun Yan, Ran Li, Yuan Mu, Wei-An Deng

**Affiliations:** ^1^Jiangsu Key Laboratory for Biodiversity and Biotechnology, College of Life Sciences, Nanjing Normal University, Nanjing, China; ^2^School of Chemistry and Bioengineering, Hechi University, Yizhou, China; ^3^College of Oceanology and Food Sciences, Quanzhou Normal University, Quanzhou, China

**Keywords:** positive selection, mitochondrial genes, flight, high-altitude adaptation, grasshoppers

## Abstract

The molecular evolution of mitochondrial genes responds to changes in energy requirements and to high altitude adaptation in animals, but this has not been fully explored in invertebrates. The evolution of atmospheric oxygen content from high to low necessarily affects the energy requirements of insect movement. We examined 13 mitochondrial protein-coding genes (PCGs) of grasshoppers to test whether the adaptive evolution of genes involved in energy metabolism occurs in changes in atmospheric oxygen content and high altitude adaptation. Our molecular evolutionary analysis of the 13 PCGs in 15 species of flying grasshoppers and 13 related flightless grasshoppers indicated that, similar to previous studies, flightless grasshoppers have experienced relaxed selection. We found evidence of significant positive selection in the genes *ATP8, COX3, ND2, ND4, ND4L, ND5*, and *ND6* in flying lineages. This results suggested that episodic positive selection allowed the mitochondrial genes of flying grasshoppers to adapt to increased energy demands during the continuous reduction of atmospheric oxygen content. Our analysis of five grasshopper endemic to the Tibetan Plateau and 13 non-Tibetan grasshoppers indicated that, due to positive selection, more non-synonymous nucleotide substitutions accumulated in Tibetan grasshoppers than in non-Tibetan grasshoppers. We also found evidence for significant positive selection in the genes *ATP6, ND2, ND3, ND4*, and *ND5* in Tibetan lineages. Our results thus strongly suggest that, in grasshoppers, positive selection drives mitochondrial genes to better adapt both to the energy requirements of flight and to the high altitude of the Tibetan Plateau.

## Introduction

Adenosine triphosphate (ATP) directly provides the free energy needed for animal locomotion (Shen et al., 2009). The electron transport chains in the mitochondria generate about 95% of this ATP; for this reason, mitochondria are known as cellular power plants (Shen et al., 2010; Raimundo, 2014). All 13 proteins encoded by mitochondria DNA are critical to the electron transport chain and to energy metabolism (Scheffler, 1998). Mitochondrial genes are thus key to the evolution of the cellular mechanisms that metabolize molecular energy (Da et al., 2008).

Flight is among the most energy-consuming methods of animal locomotion. It has thus been suggested that selective pressure of mitochondrial genes is directly related to the flight ability of birds and some mitochondrial genes undergo relaxed selective constraints in flightless birds (Shen et al., 2009). Mitochondrial genes were even targets of natural selection and allowed adaptation to the huge change in energy demand that were required during the origin of bat flight (Shen et al., 2010). Similar phenomena were also found in insects. The relaxed selection was associated with flight loss of insects (Mitterboeck and Adamowicz, 2013; Mitterboeck et al., 2017); and significant positive selection was shown on the mitochondrial protein-coding genes (PCGs) of the most recent common ancestor of Pterygota, as well as on the mitochondrial PCGs of flying insects (Yang et al., 2014; Mitterboeck et al., 2017). However, since the origin of winged insects (about 406 million years) (Misof et al., 2014), the oxygen content of the earth’s atmosphere has experienced several fluctuations. The recent high peak (32%) of atmospheric oxygen content is about 100 million years ago (Bergman et al., 2004). After that, the oxygen content continued to drop until 21% (Bergman et al., 2004). Considering that there is a proportional relationship between the amount of ATP produced by animals and the concentration of oxygen in a certain range, animals need more efficient energy metabolism when locomoting in a low oxygen environment. We speculate that in the evolution of high-oxygen environments to low-oxygen environments, in order to maintain flight capability of insects, more efficient energy metabolism mechanisms are needed. At the same time, that genes involved in energy metabolism allowed adaptation to the huge change in energy demand.

Mitochondrial evolution not only relates to mode of animal locomotion, but also relates to the adaptation of animals to high-altitude habitats (Luo et al., 2013). The Tibetan Plateau, known as the “roof of the world,” is the highest plateau on earth, with an average elevation of more than 4000 m (Zhang X.Z. et al., 2005). The harsh environment of the Tibetan Plateau poses ecological challenges to its animal inhabitants that include high levels of solar radiation, low air temperatures, and low air pressures (Zhang X.Z. et al., 2005). Animals endemic to the Tibetan Plateau have developed various adaptive mechanisms to adjust to this unforgiving environment over evolutionary time (Qiu et al., 2012; Rong et al., 2012; Zhao et al., 2013). Numerous studies have shown evidence for positive selection in the mitochondrial genes of various taxa endemic to the Tibetan Plateau, including mammals, birds, and fishes (Luo et al., 2013; Zhou et al., 2014; Ma et al., 2015; Wang et al., 2016). However, the evolutionary pressures on mitochondrial genes in insects endemic to the Tibetan Plateau remain largely unexplored.

Grasshoppers evolved from a common ancestor with flight ability (Song et al., 2015). They occupy diverse terrestrial habitats worldwide, with the exception of the polar regions (Uvarov, 1966; Kevan, 1982). Over evolutionary time, some grasshoppers, such as the desert locusts and the migratory locusts, have retained wings and developed strong flight ability; these species can fly for long periods of time and cover long distances (Liu et al., 2007). Other descendants, secondarily and independently, have lost their ability to fly, and their wings have gradually degenerated or even disappeared completely in adapting to special habitats such as the Tibetan Plateau (Huang et al., 1999). Thus, grasshoppers are among excellent models in which to explore mitochondrial gene adaptations.

In this study, we aimed to explore differences in patterns of molecular evolution of 13 mitochondrial PCGs between flying and flightless grasshoppers (the wings have deteriorated) in the process of decreasing atmospheric oxygen content and the possible role of PCGs in the adaption of grasshoppers that live in The Tibetan Plateau to high-altitude environments by selective pressure analysis. We used mitochondrial gene sequences of 33 different grasshoppers in our analysis. Theses grasshoppers separated from their last common flying ancestor approximately 140 million years ago (Song et al., 2015). Of the 33 grasshoppers, 15 are flying species, 13 flightless, and 5 Tibetan (flightless). Of these mitogenomes, 32 were previously published. The mitogenome of an additional species is sequenced by us, *Chondracris rosea*, for the first time, as this species is one of the largest known grasshopper and a strong flyer.

## Materials and Methods

### Sequencing the Mitochondrial Genome of *C. rosea*

We collected specimens of *C. rosea* from Jiuhua mountain in Anhui Province, China. We extracted total genomic DNA from the hind femur muscle of two specimens with a Wizard Genomic DNA Purification Kit (Promega, United States), following the manufacturer’s instructions. Total genomic DNA was used as a template for subsequent polymerase chain reactions (PCRs).

We amplified and sequenced the complete mitochondrial genome of *C. rosea* using general primers (Simon et al., 1994) and 16 pairs of novel primers designed for this study (Supplementary Table 1). Mitochondrial genome fragments were amplified with TaKaRa Taq (Takara, Japan) using standard PCR cycling conditions: an initial denaturation at 94°C for 5 min; followed by 35 cycles of denaturation at 94°C for 30 s, annealing at 50–60°C for 30 s, and extension at 72°C for 30–60 s; and a final elongation at 72°C for 10 min. The quality and quantity of the PCR products were tested using electrophoresis on a 1% agarose gel. We directly sequenced the PCR fragments in both directions, and large PCR products were sequenced using primer walking. Sequence data were assembled and annotated using the Staden sequence analysis package (Staden et al., 2000). We analyzed the codon usage of the 13 protein-coding mitochondrial genes with MEGA 7 (Kumar et al., 2016) and ClustalW (Thompson et al., 1994). We identified transfer RNAs with tRNA-scan SE 1.21 (Lowe and Eddy, 1997), and other genes were identified by comparison with the mitochondrial genomes of known related species, such as *Schistocerca gregaria* (Erler et al., 2010).

### Phylogenetic Analysis

In addition to *C. rosea*, we used the 32 previously published grasshopper mitogenomes from the Acridoidea and Pyrgomorphoidea available in GenBank^1^ as of January 2017. Of these, 15 were long-winged (wing is longer than the abdomen) with strong flying abilities, 13 were wingless, and 5 were Tibetan endemics and also wingless (Supplementary Table 2). We also used 1 species from the Eumastacoidea and 1 from the Tetrigoidea as outgroups (Supplementary Table 2).

We extracted the 13 PCGs from all 35 grasshopper mitogenomes based on the genome annotations in GenBank and on sequence alignments. We concatenated all 13 genes for each species, and then aligned the combined genes with Muscle (Edgar, 2004) using default settings. The multiple sequence alignment was verified by eye in MEGA 7. The best-fit partitioning scheme for our alignment was calculated using Partition Finder (Jobb et al., 2004) with the GTRGAMMA model. Based on our multiple sequence alignment, we constructed a maximum likelihood (ML) phylogenetic tree with 1000 bootstrap replicates using RAXML Blackbox on the publically available supercomputer resources at CIPRES^2^. To avoid interactions between flying and Tibetan species in the selection pressure analysis, we extracted two working topologies from our ML phylogenetic tree: a flying/flightless (F-FL) topology, which included 28 low-altitude flying (15) and flightless (13) grasshoppers and did not include any Tibetan Plateau endemics; a Tibetan/non-Tibetan (T-NT) topology, which included 18 flightless Tibetan (5) and non-Tibetan (13) grasshoppers (same as the 13 in the previous group).

### Selective Pressure Detection

**FIGURE 1:**
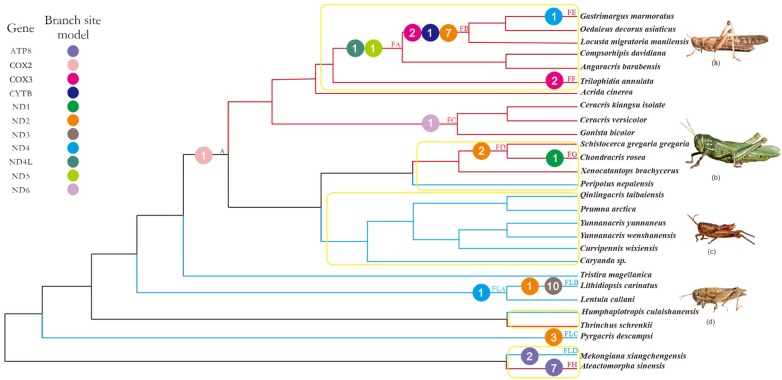
Working topology used to analyze the selective pressures on flying grasshoppers and flightless grasshoppers, extracted from the grasshopper phylogeny shown in Supplementary Figure 2. The red, blue, and black branches indicate flying, flightless, and common ancestral branches, respectively. FA-FH, FLA-FLD, and A indicate branches that detected positive selection signals. Different colored circles represent different mitochondrial genes. The number within each colored circle represents the number of positive selection sites detected on the gene. Branches within the yellow box are pairs of branches selected for branch model analysis. (a) *Locusta migratoria manilensis*, (b) *Chondracris rosea*, (c) *Yunnanacris yunnaneus*, (d) *Lentula callani*. Photo credits: (a,b) (https://www.baidu.com/), (c) Huimeng Lu, and (d) (http://orthoptera.speciesfile.org/HomePage/Orthoptera/HomePage.aspx).

**FIGURE 2:**
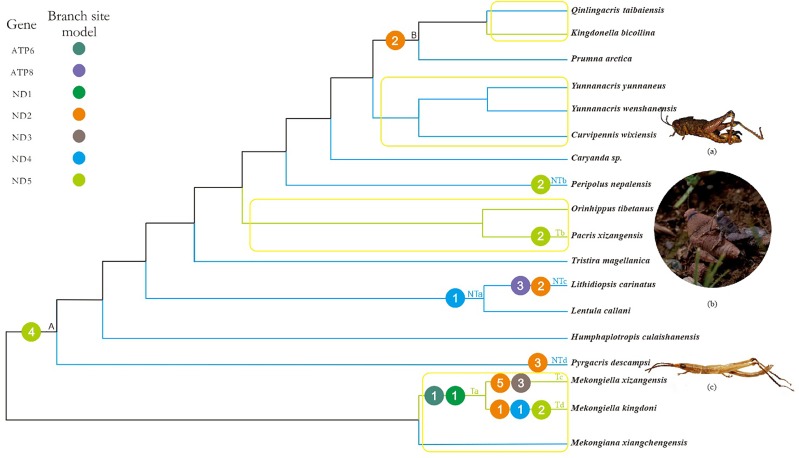
Working topology used to analyze the selective pressures on Tibetan and non-Tibetan grasshoppers, extracted from the grasshopper phylogeny shown in Supplementary Figure 2. The green, blue, and black branches indicate Tibetan, non-Tibetan, and common ancestral branches, respectively. Ta-Td, NTa-NTd and A, B indicate branches that detected positive selection signals. The different colored circles represent different mitochondrial genes. The number within each circle represents the number of positive selected sites detected on the gene. Branches within the yellow box are pairs of branches selected for branch model analysis. (a) *Peripolus nepalensis*, (b) *Pacris xizangensis*, (c) *Pyrgacris descampsi.* Photo credits. (a,c) (http://orthoptera.speciesfile.org/HomePage/Orthoptera/HomePage.aspx); (b) Yulong Zhang.

We tested for selective pressure on homologous mitochondrial PCGs by comparing the ratio of non-synonymous to synonymous substitutions (ω = dN/dS). The ω was estimated using the codon-based ML method (CODEML) in PAML 4.7 (Yang, 2007). To detect the variation of selective pressures among different grasshopper lineages, we used two PAML models. The free-ratio model estimates independent ω values for each branch (Yang, 1998); here, we used only ω values for the terminal branches and focused only on the rate of accumulation of mutations (ω) between modern species and their most recent reconstructed ancestors. The ω values of all terminal branches of the flying or Tibetan lineage were classified into one group and those of the flightless or non-Tibetan lineage were classified into another group. We then used the Wilcoxon rank sum test to determine whether the ω values differed significantly between the groups analyzed (i.e., flying versus flightless; Tibetan versus non-Tibetan). The branch models test whether heterogeneous selective pressures act on specific branches and lineages (Yang, 1998). Here, we used the two-ratio and the three-ratio branch models: the two-ratio branch model allows a background ω ratio and a different ω ratio for the lineage of interest. In our analysis, the selected paired lineages are used as foreground branches (ω_1_) and the remaining branches are used as background branches (ω_0_) (Figures 1, 2). The three-ratio branch model allows one ω value for deep branches, a second ω value for the external branches of the lineage of interest, and a third ω value for the external branches of other lineages. The three-ratio branch model avoids result bias because ω value estimates are higher for tip lineages (Ho et al., 2007). This is particularly relevant for traits like flight that tend to be lost unidirectionally, resulting in the reconstruction of deeper nodes as trait-having (Hovmöller et al., 2002; Stone and French, 2003; Mitterboeck and Adamowicz, 2013). In our analysis, based on selected paired lineages, flying lineages or Tibetan lineages were coded 1 branch rate (ω_1_), flightless lineages or non-Tibetan lineages were coded another branch rate (ω_2_), the remaining branches are coded as background branch (ω_0_) (Figures 1, 2). Likelihood ratio tests between three-rate trees and two-rate trees were used to test for significant ω differences between target lineages and sister lineages.

To quantify the probability of positive selection on each site in each gene across all grasshopper sequences, we implemented site models (M1 and M2, M8a and M8), where ω was allowed to vary among sites (Yang, 2007). Finally, the branch-site model (Zhang J. et al., 2005) tests for signals of positive selection that act over short periods of evolutionary time and on just a few sites in the gene. The alternative model of positive selection (MA; 0 < ω0 < 1, ω1 = 1, ω2 ≥ 1) and the null model of neutral evolution (MA0; ω2 = 1) in the branch-site test were used to detect selective pressure on each branch. We used this model to identify positive selection on a small number of genomic sites along all grasshopper lineages.

To determine the model that best fit our data, we used the likelihood ratio test (LRT) to compare each pair of models. We tested the differences among LRTs using Chi square distributions, where twice the difference of the log likelihood between the pair of models (2ΔlnL) was asymptotic to the Chi square distribution. The degree of freedom for each chi square distribution was equal to the difference in the number of free parameters between the two models. We calculated the posterior probability that each site class of the foreground lineages was subject to positive selection using the empirical Bayes method implemented in CodeML in PAML 4.7 (Zhang J. et al., 2005).

In addition, we used fixed-effect likelihood (FEL) and fast unconstrained Bayesian approximation (FUBAR), as implemented on the Datamonkey website, to detect positive selection; these tests compute synonymous and non-synonymous substitutions at each codon position (Pond and Frost, 2005). We considered sites with a FEL significance < 0.1 or a FUBAR posterior probability > 0.9 as candidates for selection. In this way, we avoided the danger of a small number of sequences leading to high false positive rates.

### Structural Analysis

To gain insight into the functional significance of the putatively selected sites, we mapped these sites onto the three-dimensional (3D) structures of the proteins. We predicted 3D gene structures using the homology modeling software provided by the I-TASSER server60 (Zhang, 2008). The protein sequences of positively selected genes were derived from *Locusta migratoria manilensis* mitochondrial genome, which were obtained from GenBank. We obtained functional information for the genes putatively identified as positively selected from UniProt^3^.

## Results

### General Characteristics of the *C. rosea* Mitogenome

The complete mitogenomic sequence of *C. rosea* has been deposited in GenBank (Accession No. NC_019993). The mitogenome was a circular molecule 15,646 bp long, with typical arthropod mitogenomic content: 13 PCGs, 22 tRNAs, 2 ribosomal RNAs, and an A/T-rich region (Supplementary Figure 1 and Supplementary Table 3), and 10 non-coding regions. The j-strand of the *C. rosea* mitogenome was 31.3% T, 42.5% A, 15.2% C, and 11.0% G; the total A+T content was 73.8%. The largest non-coding region was 760 bp long and was located between *12S rRNA* and *tRNA^Ile^*. All protein-coding sequences had a ATN codons. We identified canonical initiation codons (ATA or ATG) in 10 PCGs (*ND2, COX2, ATP6, COX3, ND3, ND6, ND4, ND4L, ATP8*, and *ND1*). Three genes (*COX1, CYTB*, and *ND5*) had ATT or ATC start codons.

The 22 tRNAs in the *C. rosea* mitogenome were identified based on their secondary structures and the primary sequences of the corresponding anticodon. Except for *tRNA^Ser-AGN^*, which lacks a dihydrouridine (DHU) arm, all of tRNAs formed clover-leaf structures. There was an 8 bp overlap between the *tRNA^Trp^* and *tRNA^Cys^* genes. The two ribosomal RNAs identified in the *C. rosea* mitogenome, 12S rRNA and 16S rRNA, were located between *tRNA^V al^* and the A+T-rich region, and between *tRNA^Leu(CUN)^* and *tRNA^V al^*, respectively.

### Working Topology Construction

Our phylogenetic analysis recovered a well-supported Pyrgomorphoidea (100% bootstrap) and a moderately well-supported Acridoidea (77% bootstrap) (Supplementary Figure 2). As the phylogenetic relationships we recovered were congruent with two recent robust phylogenies of the Orthoptera based on complete mitochondrial genomes and nuclear genes (Song et al., 2015; Zhao et al., 2018), our phylogenetic tree was suitable for use as the basis for two working topologies. Our F-FL topology included 15 flying species and 13 flightless species (2 from the Pyrgomorphoidea and 26 from the Acridoidea; Figure 1). None of these species were native to the Tibetan Plateau. Our N-NT topology included 5 Tibetan and 13 non-Tibetan species (3 from the Pyrgomorphoidea and 15 from the Acridoidea; Figure 2). None of these species fly.

### Selective Pressures on Flying and Flightless Grasshoppers

**FIGURE 3:**
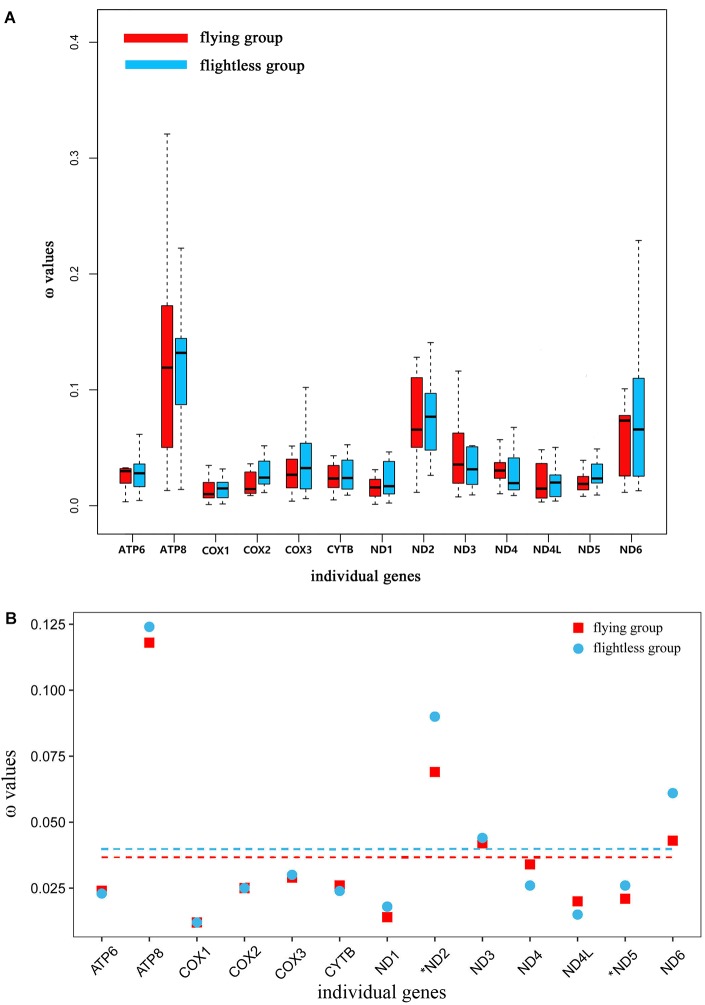
**(A)** Ratio of non-synonymous to synonymous substitutions (ω) in the 13 protein-coding mitochondrial genes of flying and flightless grasshoppers based on free-ratio model. Boxes include 50% of values; ω is not significantly different between the flying and flightless grasshoppers for any gene. *ATP6, P* = 0.93; *ATP8, P* = 0.83; *COX1, P* = 0.63; *COX2, P* = 0.36; *COX3, P* = 0.87; *CYTB, P* = 0.50; *ND1, P* = 0.87; *ND2, P* = 0.50; *ND3, P* = 0.11; *ND4, P* = 0.24; *ND4L, P* = 0.76; *ND5, P* = 0.12; *ND6, P* = 0.72. **(B)** Ratio of non-synonymous to synonymous substitutions (ω) in the 13 protein-coding mitochondrial genes of flying and flightless grasshoppers based on 3 vs. 2 ratio model. Genes with a significant difference in rates are marked with an asterisk; in all two cases, the dN/dS ratio is higher in the flightless lineages than in their flight-capable counterparts. Dashed lines signify the mean ω values; flying: 0.036 and flightless: 0.040.

In the free-ratio model, the ω values for the 13 PCGs were no significant difference in flying grasshoppers as compared to flightless grasshoppers (Figure 3A). We used the three-ratio model and the two-ratio model to calculate selective pressures acting on flightless grasshoppers and flying grasshoppers to further examine the difference between them. LRT tests indicated that, except for *ND2* and *ND5*, the two-ratio model fit our data significantly better than the three-ratio model for 11 genes. The ω values for eight genes *ATP8, COX2, COX3, ND1, ND2, ND3, ND5*, and *ND6* were bigger in the flightless grasshoppers as compared to the flying grasshoppers; *ND2* and *ND5* was significant bigger (Figure 3B and Supplementary Table 4).

To determine if individual gene codons were subject to positive selection, we used two pairs of site models (M1 vs. M2 and M8a vs. M8). The M8 model identified one positively selected site on the *ND2* gene (Supplementary Table 5). Significant evidence of positive selection was also found by the FEL and FUBAR models. FEL identified nine positively selected codons in five genes (*ND2, ND4, ND4L, ND5*, and *ND6*) (significance < 0.1), and FUBAR identified 12 positively selected codons in seven genes (*ATP8, COX2, COX3, ND2, ND4, ND4L, ND6*) (FUBAR posterior probability > 0.9; Supplementary Table 6).

Using the more stringent branch-site model, we detected signals of positive selection in 13 branches and 11 genes; 44 amino acid sites were found to be under positive selection (posterior probability ≥ 95%; Supplementary Table 7). Of these, nine genes (*ATP8, COX3, CYTB, ND1, ND2, ND4, ND4L, ND5, ND6*) and 26 amino acid sites were identified on the flying branches, while four genes (*ATP8, ND2, ND3, ND4*) and 17 amino acid sites were identified on the flightless branches (Figure 1). The positively selected genes and sites on flying branches were found on branch FA (two genes and two sites), branch FB (3 genes and 10 sites), branch FC (one gene and one site), branch FD (one gene and two sites), branch FE (one gene and one site), branch FF (one gene and two sites), branch FG (one gene and one site), and branch FH (one gene and seven sites). The positively selected on flightless branches were found on branch FLA (one gene and one site), branch FLB (2 genes and 11 sites), branch FLC (one gene and three sites), and branch FLD (one gene and two sites) (Figure 1 and Supplementary Table 7).

Combined with the above four models used to analyze positive selection, the seven positive selected genes (*ATP8, COX3, ND2, ND4, ND4L, ND5*, and *ND6*) of flying branches were found by the branch-site model and at least one other method simultaneously, while only three positive selected PCGs (*ATP8, ND2*, and *ND4*) on flightless branches.

We next investigated the functional domains of the seven positively selected PCGs on flying branches to determine the functional significance of the putative positively selected sites. We localized most of the positively selected sites in or close to the functional regions of the proteins encoded by these seven genes. Indeed, 19 of the positively selected sites were located within the protein transmembrane domain of the encoding genes, and 13 positively selected sites were located in other protein domain of the corresponding genes (Supplementary Figure 3 and Supplementary Table 8).

### Selective Pressures on Tibetan and Non-Tibetan Grasshoppers

**FIGURE 4:**
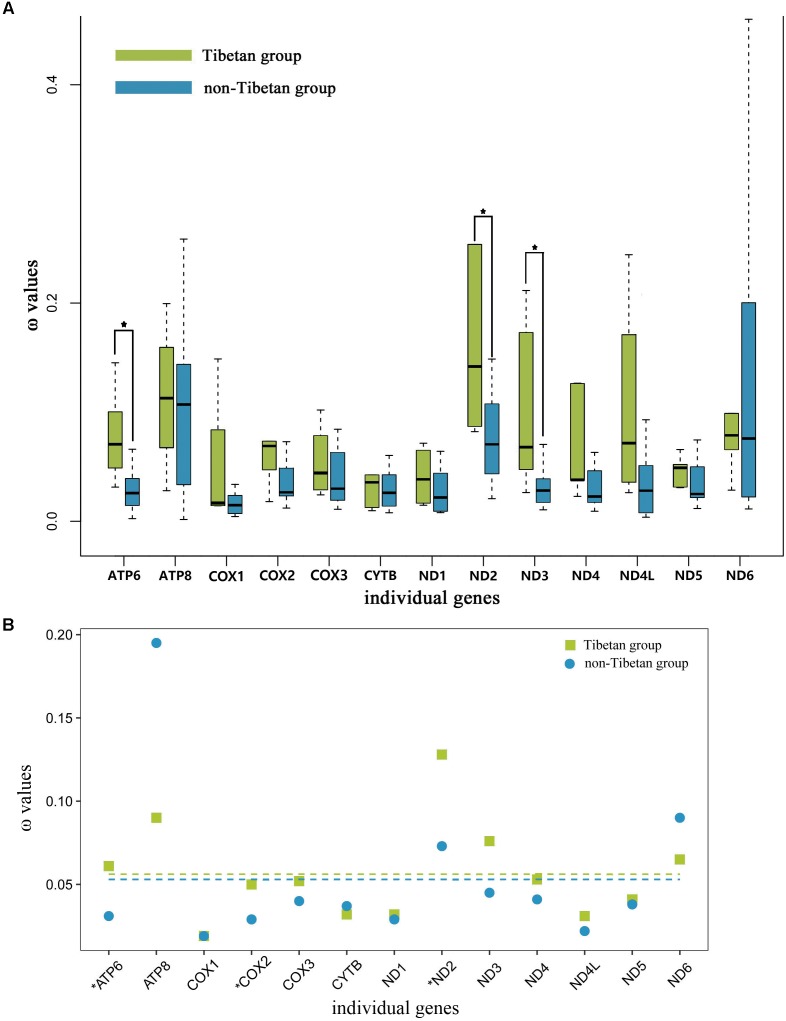
**(A)** Ratio of non-synonymous to synonymous substitutions (ω) in the 13 protein-coding mitochondrial genes of Tibetan and non-Tibetan grasshoppers based on free-ratio model. Boxes include 50% of values; ^∗^ indicates a significant difference in ω between Tibetan and non-Tibetan grasshoppers for that gene. *ATP6, P* = 0.01; *ATP8, P* = 0.66; *COX1, P* = 0.81; *COX2, P* = 0.08; *COX3, P* = 0.08; *CYTB, P* = 0.52; *ND1, P* = 0.08; *ND2, P* = 0.03; *ND3, P* = 0.03; *ND4, P* = 0.07; *ND4L, P* = 0.46; *ND5, P* = 0.18; *ND6, P* = 0.66. **(B)** Ratio of non-synonymous to synonymous substitutions (ω) in the 13 protein-coding mitochondrial genes of Tibetan and non-Tibetan grasshoppers based on 3 vs. 2 ratio model. Genes with a significant difference in rates are marked with an asterisk; in all three cases, the dN/dS ratio is higher in the Tibetan lineages than in their non-Tibetan counterparts. Dashed lines signify the mean ω values; Tibetan: 0.056 and non-Tibetan: 0.053.

The free-ratio model indicated that all of the 13 mitochondrial PCGs had larger ω values in the Tibetan grasshoppers than in the non-Tibetan grasshoppers. These differences were significant in three of these genes: *ATP6* (*p* = 0.01), *ND2* (*p* = 0.03), and *ND3* (*p* = 0.03) (Figure 4A). Using the three-ratio model and the two-ratio model, we calculated selective pressures acting on Tibetan grasshoppers and non-Tibetan grasshoppers to further examine the difference between them. LRT tests indicated that, except for *ATP6, COX2* and *ND2*, the two-ratio model fit our data significantly better than the three-ratio model for 10 genes. The ω values for nine genes *ATP6, COX2, COX3, ND1, ND2, ND3, ND4, ND4L*, and *ND5* were bigger in Tibetan grasshoppers as compared to non-Tibetan grasshoppers; *ATP6, COX2*, and *ND2* was significant bigger (Figure 4B and Supplementary Table 9).

We next used two pairs of site model (M1 vs. M2 and M8a vs. M8) to determine whether the individual codons of each gene were under positive selection. Neither model identified any genes nor sites under positive selection. We then searched for evidence of positive selection with the FEL and FUBAR models. FEL identified 10 positively selected codons in seven genes (*ATP6, COX2, COX3, ND2, ND4, ND5*, and *ND6*) (FEL significance < 0.1), while FUBAR identified 10 positively selected codons in five genes (*ATP6, COX3, ND2, ND3*, and *ND6*) (FUBAR posterior probability > 0.9; Supplementary Table 10).

Based on the branch-site model, we detected signals of positive selection along 10 branches, in seven genes and at 33 amino acid sites (posterior probability ≥ 95%; Supplementary Table 11). Of these positively selected genes and amino acid sites, six genes (*ATP6, ND1, ND2, ND3, ND4*, and *ND5*) and 16 amino acid sites were identified on Tibetan branches, while four genes (*ATP8, ND2, ND4*, and *ND5*) and 11 amino acid sites were identified on non-Tibetan branches (Figure 2). The positively selected genes and sites on Tibetan branches were found on branch Ta (two genes and two sites), branch Tb (one gene and two sites), branch Tc (two genes and eight sites), and branch Td (three genes and four sites). The positively selected genes and sites on non-Tibetan branches were found on branch NTa (one gene and one site), branch NTb (one gene and two sites), branch NTc (two genes and five sites), and branch NTd (one gene and three sites) (Figure 2 and Supplementary Table 11).

Combined with the above four models used to analyze positive selection, the five positive selected genes (*ATP6, ND2, ND3, ND4*, and *ND5*) of Tibetan branches were found by the branch-site model and at least one other method simultaneously, while only three positive selected PCGs (*ND2, ND4*, and *ND5)* on non-Tibetan branches.

The functional domains of five positively selected PPGs on Tibetan branches were examined to determine the functional significance of the putative positively selected sites. We found that most of the putatively positively selected sites were located in or close to the functional regions of the proteins encoded by the five PCGs. In four of these PCGs (*ND2, ND3, ND4*, and *ND5*), nine positively selected sites were located within the protein transmembrane domain of the encoding gene, and 11 positively selected sites were located in other protein domain of the corresponding genes (Supplementary Figure 3 and Supplementary Table 12).

## Discussion

### The Mitogenome of *C. rosea*

The mitogenome of *C. rosea* was 21 bp longer than that of *Schistocerca gregaria* (Erler et al., 2010), indicating that mitogenomes of related species may differ in length. It is possible that the large non-coding region in the *C. rosea* genome located between *12S rRNA* and *tRNA^Ile^* might have a regulatory function (Lee et al., 2006). Consistent with the mitochondrial genomes of other arthropods, mitogenome of *C. rosea* contained 13 PCGs (Clary and Wolstenholme, 1985) (Supplementary Figure 1 and Supplementary Table 3). The start codons of *COX1, CYTB*, and *ND5* in *C. rosea*, ATT and ATC, have also been reported in other species (Lessinger et al., 2000). The stop codons in the *C. rosea* genome are similar to those described in other insects, and may have been created by polyadenylation, as has been suggested in other animal phyla (Castresana et al., 1998; Nardi et al., 2001). Except for *tRNA^Ser-AGN^*, which lacks a DHU arm, all *C. rosea* tRNAs formed cloverleaf structures, and their anticodons were similar to those found in other orthoptera insects (Zhao et al., 2018). Thus, it is clear that the mitogenome of *C. rosea* is relatively highly conserved.

The *C. rosea* mitogenome had an A/T content (73.8%) consistent with that of other arthropods (69.5 to 84.9%) (Crozier and Crozier, 1993; Dotson and Beard, 2001), but higher than that of *S. g. gregaria* (73.1%), implying that the mitogenomes of related species may differ slightly with respect to A/T content. Interestingly, the 8 bp overlap between *tRNA^Trp^* and *tRNA^Cys^* has a known function: this overlap causes the two genes to produce separate transcripts with opposite directionality (Lessinger et al., 2000).

### Positive Selection and the Evolution of Grasshopper Flight

The evolution of flight has been critical to the success of winged insects; flying insects such as locusts are the most successful of the terrestrial arthropods because of their ability to access diverse food sources by flying long distances (Mayhew, 2003; Grimaldi and Engel, 2005; Liu et al., 2007). Flight is energetically costly. For example, the winged morph of the pygmy grasshopper (*Tetrix subulata*) consumes significantly more energy than the wingless morph (Lock et al., 2006). Most of the energy required for flight is provided by mitochondrial electron transport chain (Shen et al., 2009, 2010). Mitochondrial genes encode all of the complexes related to oxidative phosphorylation except for succinate dehydrogenase (complex II) (Scheffler, 1998; Carroll et al., 2009; McKenzie et al., 2009). Therefore, positive and relaxed selection of mitochondrial genes were associated with flight evolution and loss of insects (Mitterboeck and Adamowicz, 2013; Yang et al., 2014; Mitterboeck et al., 2017). This fully demonstrates that the molecular evolution of mitochondrial genes responds to changes in the energy requirements of insects.

Changes in atmospheric oxygen content will inevitably affect the energy needs of the grasshoppers. Therefore, the evolution of atmospheric oxygen content from high to low should affect the molecular evolution of mitochondrial genes in grasshoppers. This study tested for molecular evolution trends of mitochondrial protein-encoding genes of flying and flightless grasshoppers during their evolution from a high-oxygen (32%) environment 100 million years ago to the current low-oxygen (21%) (Bergman et al., 2004). Based on the free-ratio model that has been well-applied in bird research (Shen et al., 2009), we did not find significant differences between the flying and flightless grasshoppers (Figure 3A). This may be because the mitochondrial gene evolution rate of grasshoppers was relatively slow and we focused only on the rate of accumulation of slightly deleterious mutations (dN/dS) between modern species and their most recent reconstructed ancestors. However, the increased ω values were found on the flightless lineages by using three-ratio versus two-ratio model (Figure 3B and Supplementary Table 4). This indicated that, although the atmospheric oxygen content was reduced, the flightless grasshoppers have experienced relaxed selection in mitochondrial genes due to flight losses and was accord with previous observations of insect orders (Mitterboeck and Adamowicz, 2013; Mitterboeck et al., 2017). On the contrary, like birds, the flying grasshoppers have experienced stronger evolutionary constraints to eliminate deleterious mutations, and maintain efficient energy metabolism (Shen et al., 2009).

Considering that 100 million years ago, the atmospheric oxygen content was almost 11% higher than it is now (Bergman et al., 2004), flying grasshoppers may need more efficient mitochondria to maintain the flight capacity. Therefore, mitochondrial genes of flying grasshoppers may have evolved mechanisms to adapt to increased energy demands. As positive selection typically acts only on a few sites for a short period of evolutionary time, continuous negative selection that occurs on most sites usually swamps any signals of positive selection in a gene sequence (Zhang J. et al., 2005). So, we tested for positive selection with branch-site models, which detect variations in selective pressure both at individual amino acid sites and along lineages; branch-site models are powerful tools for distinguishing positive selection from purifying selection (Zhang J. et al., 2005). We found evidence of significant positive selection at 44 amino acid sites across 11 mitochondrial PCGs in F-FL lineage (Figure 1 and Supplementary Table 7). Almost twice as many positively selected genes and amino acid sites were detected on flying branches as compared to flightless branches. Most of the positively selected genes on flying branches were found on branches FA and FB (Figure 1). This is consistent with evidence of flight ability because branches FA and FB fall within the Oedipodinae; species in this subfamily have the strongest flight ability of all the grasshopper taxa. The species *Schistocerca gregaria gregaria* and *C. rosea* are also strong fliers, so it is not surprising that we found evidence of positive selection on the branch ancestral to these species (FD) and the terminal branch (FG). Seemingly, the selective strength has some correlation with flight ability, i.e., relatively more genes were subjected to positive selection among stronger flying lineages. In addition, we also identified a few positive selection acting on flightless branches. They were mainly identified on branch FLB (*Lithidiopsis carinatus*) and branch FLA (the least ancestor of FLB) (Figure 1). *L. carinatus* live in the more arid and desert regions of South and South West Africa. In order to adapt to environment, its external morphology has come to resemble sand, small rocks, and pebbles (Otte, 2007). Because previous studies have proposed that climate can drive the differentiation of human mitochondrial DNA (Ruiz-Pesini et al., 2004), we speculate the positive selection of branches FLB and FLA may be related to adaptation to the arid environment. In short, the identification of many genes as positively selected suggested that episodic positive selection has acted on flying grasshopper mitochondrial PCGs.

In order to make the evidence of positive selection more robust, besides the branch site model, the site model, FEL and FUBAR models were also employed. Combined with the results of four models, seven positive selected genes (*ATP8, COX3, ND2, ND4, ND4L, ND5*, and *ND6*) on the flying branches were detected by branch-site model and at least one other method simultaneously. The seven PCGs play an important role in mitochondrial oxidative phosphorylation: *ND2, ND4, ND4L, ND5* and *ND6*, are subunits of NADH dehydrogenase (mitochondrial complex I), and NADH hydrogenate begins oxidative phosphorylation process. Complex I, the largest and most complicated proton pump of the respiratory chain, couple electron transfer from NADH to ubiquinone with transmembrane proton pumping contributing to the proton motive force used for ATP synthesis (Wirth et al., 2016). It plays a central role in cellular energy metabolism and more than one-third of mitochondrial energy production is driven by a gradient of protons across the mitochondrial membrane created by the pumping action of complex I (Dröse et al., 2011). *ND2, ND4*, and *ND5* have been discussed as prime candidates for harboring the proton pumps (Mathiesen and Hägerhäll, 2002). This might explain why we detected more evidence of positive selection in complex I than in other complexes. Cytochrome c oxidase (Complex IV) is directly involved in electron transfer and proton translocation, whereas *COX3*, part of the catalytic core of Complex IV, may act as a regulator (Zhang H. et al., 2013). *ATP8* is part of ATP synthase (Complex V), where it plays an essential role in the final assembly of ATPase (Zhang H. et al., 2013). The positively selected sites observed in the seven PCGs on flying branches were localized in or near functional regions, based on the crystal structures of the encoding genes (Supplementary Figure 3 and Supplementary Table 8). This indicated that the adaptive evolution of the mitochondrial PCGs of flying grasshoppers is ongoing, acting to increase energy supply and thus improve flight ability.

Collectively, our results indicate that positive selection allows the mitochondrial genes of flying grasshoppers to adapt to the increased energy requirements required to maintain flight during the continuous reduction of atmospheric oxygen content.

### Positive Selection and the Adaptation to High-Altitudes

Various adaptive responses have allowed grasshoppers survive the extreme environment of the Tibetan plateau, including wing degeneration and loss (Yin, 1984). However, the role of mitochondrial genes in encoding these adaptive responses remains largely unexplored, not just in grasshoppers, but also across all invertebrates.

To understand the role of mitochondrial genes in high-altitude adaptation, we compared the selective pressures acting on 13 mitochondrial PCGs in Tibetan grasshoppers to those in non-Tibetan grasshoppers. We tested whether the mitochondrial DNA of Tibetan and non-Tibetan grasshoppers experienced different selective pressures by calculating the ω values associated with terminal branches based on free ratio model. The 13 mitochondrial PCGs had greater ω values on Tibetan branches than on non-Tibetan branches. In particular, the ω values of *ATP6, ND2*, and *DN3* were significantly larger in Tibetan grasshoppers than in non-Tibetan grasshoppers (Figure 4A), implying that Tibetan lineages have accumulated more non-synonymous mutations over evolutionary time. Similarly, the three- and two -ratio branch models indicated that, in nine mitochondrial PCGs, Tibetan lineages had larger average ω values than did non-Tibetan lineages (Figure 4B and Supplementary Table 9).

In principle, a high ω value might be caused either by positive selection or by relaxed functional constraints, and it is difficult to distinguish between these two possibilities based exclusively on the ω value (Li et al., 2017). Positive selection is more likely to lead to the fixation of beneficial non-synonymous mutations, whereas relaxed functional constraints are expected to decrease the degree of purifying selection, which can lead to the fixation of deleterious mutations (Li et al., 2017). Given the importance of mitochondrial oxidative phosphorylation to aerobic organisms, grasshoppers endemic to the Tibetan plateau must have evolved mechanisms to cope with the harsh environment. We therefore deduced that positive selection may have occurred on some mitochondrial genes in Tibetan lineages over their evolutionary history. As expected, branch-site model detected strong signals of positive selection in 33 amino acid sites in seven PCGs (posterior probability ≥ 95%) on 10 branches (Figure 2 and Supplementary Table 11). Based on the distribution of PCGs in T-NT lineage identified as positively selected by branch-site model, 1.5 times more genes and amino acid sites were positive selected on Tibetan branches, as compared to non-Tibetan branches. However, 2.6 times more non-Tibetan species than Tibetan species were included in the T-NT lineage. Therefore, the ratio of positively selected genes and sites should be greater than 1.5 between Tibetan and non-Tibetan branches. The genes under positive selection on Tibetan branches were mainly found on branches Ta, Tc, Td, and Tb. No positive selection genes were detected on the other two Tibetan species, which may be due to adaptive evolution frequently occurs in episodic bursts, localized to a few sites in a gene, and to a small number of lineages in a phylogenetic tree (Kosakovsky Pond et al., 2011); This is similar to the adaptive evolutionary analysis of mitochondrial genes in the Tibetan birds (Zhou et al., 2014). In addition, positive selection was identified on the branch NTb (*Peripolus nepalensis*) and NTd (*P. descampsi*). *Peripolus nepalensis* may be related to the distribution in the southern foothills of the Tibetan Plateau and *P. descampsi* is endemic to Reunion Island with the highest mountain at 3,070 m above sea level (Hugel, 2005), so their positive selection may be the adaptation to high altitude environment. The PCGs under positive selection on branches NTa and NTc (*L. carinatus*) was similar to the distribution of positively selected PCGs of F-FL lineage.

Combined with the results of the branch site and FEL and FUBAR models, five positive selected PCGs (*ATP6, ND2, ND3, ND4*, and *ND5*) on Tibetan branches were detected by the branch-site model and at least one other method simultaneously. There is thus strong evidence that these PCGs have been subject to positive selection. Here, the positively selected sites in five genes were located in or near functional regions, based on the crystal structures of the encoding genes (Supplementary Figure 3 and Supplementary Table 12). This indicated that adaptive evolution of Tibetan grasshoppers mitochondrial PCGs is ongoing, acting to increase energy and heat supply to cope with hypoxia and low temperatures. A previous physiological study showed that the mechanisms regulating mitochondrial cytochrome c oxidase in high-altitude migratory locusts were adapted to the persistent hypoxic environment of the Tibetan Plateau (Zhang Z.Y. et al., 2013). Although we did not detect evidence of positive selection pressure on the grasshopper cytochrome c oxidase genes, this may have been due to differences in the mechanisms used to adapt to hypoxia and low temperature between the wingless Tibetan grasshoppers and the high-altitude, strong-flying migratory locust.

Positive selection on nine genes (*ATP6, ATP8, COX1, COX2, CYTB, ND2, ND3, ND5*, and *ND6*) has been shown in mammals endemic to the Tibetan plateau including humans, antelope, horses, pika, and monkeys (Xu et al., 2005, 2007; Luo et al., 2008, 2013; Yu et al., 2011; Gu et al., 2012). Similarly, three PCGs (*ATP6, ND2*, and *ND4*) are positively selected in Tibetan birds (Zhou et al., 2014), and 12 PCGs (*ATP6, ATP8, COX1, COX2, COX3, CYTB, ND1, ND2, ND3, ND4, ND4L*, and *ND5*) are positively selected in Tibetan fish (Ma et al., 2015; Wang et al., 2016). In brine shrimp, *ATP6* may have been subject to strong selective pressure to adapt to high-altitude habitats (Zhang H. et al., 2013). These studies, in combination with our results, suggested that *ATP6* is most important PCG for adaption to high altitude environments, in addition to several subunits of NADH dehydrogenase (complex I). *ATP6* is a subunit of complex V, which, together with other membrane integral proteolipid subunits, forms the proton channel of mitochondrial ATPase, and plays an essential role in the final assembly of ATPase (Zhang H. et al., 2013). This might explain why ATP6 is subjected to increased selective pressure during adaptation to high altitude environments, but more evidence, including functional experiments, is needed to validate this conjecture.

## Conclusion

Our report, using grasshoppers as a model, first explored the molecular evolutionary mechanisms of insect mitochondrial genes in response to the reduction of atmospheric oxygen content and high altitude environments. Our results clearly indicated that positive selection drives adaptive evolution in mitochondrial genomes, both with respect to flight and with respect to survival in challenging environments. Although in this study we have implemented a full selective pressure analysis, these results need to be further validated in functional experiment of mitochondrial genes.

## Author Contributions

X-DL and G-FJ conceived and designed the experiments and wrote the paper. G-FJ, X-DL, and RL performed the experiments. X-DL, YM, LY, and W-AD collected and analyzed the data. X-DL, G-FJ, RL, and YM contributed reagents, materials, and analysis tools. All authors read and approved the final manuscript.

## Conflict of Interest Statement

The authors declare that the research was conducted in the absence of any commercial or financial relationships that could be construed as a potential conflict of interest.

## Footnotes

1 https://www.ncbi.nlm.nih.gov/

2 https://www.phylo.org/

3 http://www.uniprot.org/
