# Supplementary material for: Positive Selection Drove the Adaptation of Mitochondrial Genes to the Demands of Flight and High-Altitude Environments in Grasshoppers
Source: Front Genet. 2018 Dec 5;9:605. doi: 10.3389/fgene.2018.00605 (PMC6290170; doi:10.3389/fgene.2018.00605)
Supplement: Supplementary file 1 [file Table_1.docx]

Supplementary Material

Positive selection drove the adaptation of mitochondrial genes to the demands of flight and high-altitude environments in grasshoppers

**Xiao-Dong** **Li^1,2^ ·** **Guo-Fang** **Jiang^1,3*^** **· Li-Yun** **Yan^1^ ·** **Ran** **Li****^1^ ·** **Yuan** **Mu****^1^** **· Wei-An** **Deng^2^**

^1^ Jiangsu Key Laboratory for Biodiversity and Biotechnology, College of Life Sciences, Nanjing Normal University, Nanjing 210023, China.

^2^ School of Chemistry and Bioengineering, Hechi University, Yizhou 546300, China.

^3^ College of Oceanology and Food Sciences, Quanzhou Normal University, Quanzhou 362000, China.

**Correspondence** Guo-Fang Jiang

cnjgf1208@163.com


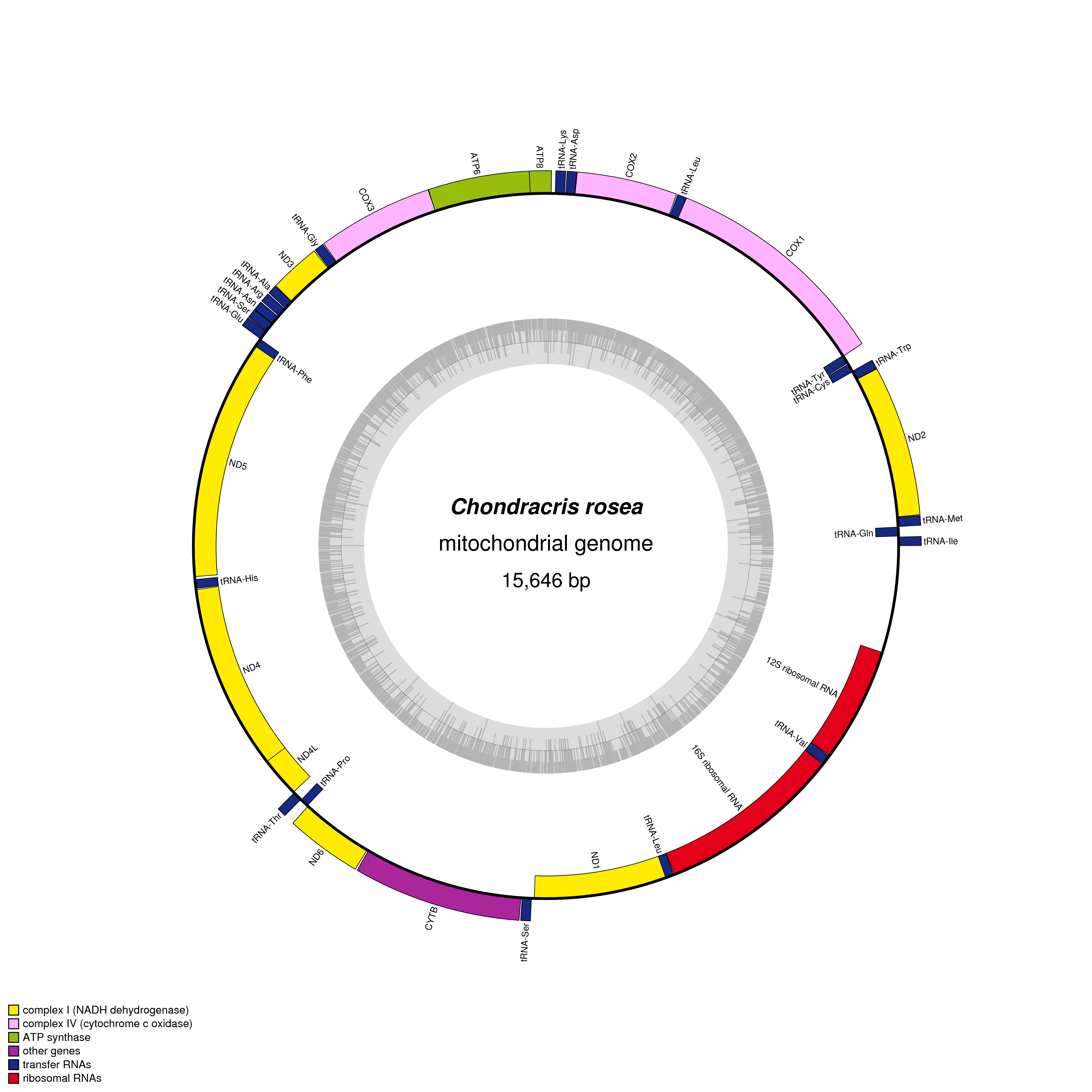


**FIGURE. 1** Map of the mitochondrial genome of *Chondracris rosea*


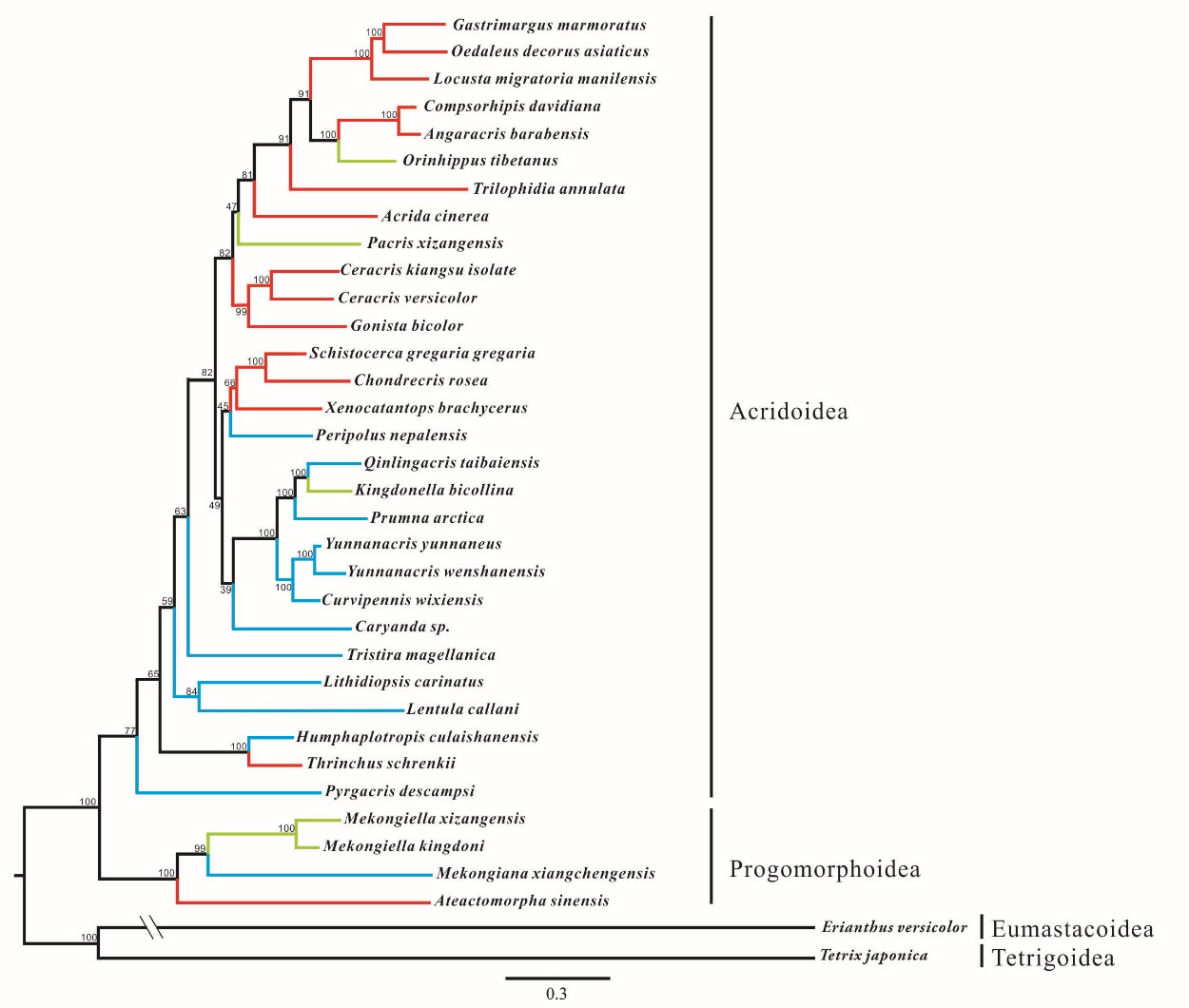


**FIGURE 2** Maximum Likelihood tree of all 13 protein-coding genes in the mitochondrial genomes of 33 grasshopper species. The red, blue, and green branches indicate flying, flightless (non-Tibetan), and Tibetan branches, respectively. Numbers at each node show bootstrap percentages. *Erianthus versicolor* and *Tetrix japonica* were used as outgroups.

**Table 1** Novel primers designed for the amplification and sequencing of the unassembled fragments of the *Chondracris rosea* mitochondrial genome

| Primer^a^ | Location | Sequence (5′→3′) |
| --- | --- | --- |
| F1 | 4 | GGAGTGCCTGACTAAAGGGTT |
| R1 | 1563 | CCAATTAATGATCCTGGTTCACC |
| F2 | 1440 | GGTCAACAAATCATAAAGATATTGG |
| R2 | 2140 | TAAACTTCAGGGTGACCAAAAAATCA |
| F3 | 5124 | GCCTCAGGAGTTACAATTACATGAGC |
| R3 | 6092 | CAGTAATACGCCTCTTTGGCTTC |
| F4 | 6004 | CCATCGCATTCAGTTTCGACC |
| R4 | 6901 | CTACTGGTTTAACGGCTTCATATTC |
| F5 | 6744 | TAATCTACCTCCAAATACAGCAAC |
| R5 | 7909 | GAATGGTTCCATTGTTAT |
| F6 | 7373 | CACCAGCTGTAACAAGAGTAGAAG |
| R6 | 8697 | GGTTATCTTTAGGTCTTTCTGGTGG |
| F7 | 8529 | CCAGATGAACATAAGCCATGACC |
| R7 | 9410 | TTGTTGATGGTTGGTCCATTC |
| F8 | 9390 | GAATGAACCAACCATCAACAAT |
| R8 | 10856 | GTAGCTCCTCAGAAGGATATTTGTCC |
| F9 | 10616 | GAAGCGTAGTCCATATTTGTCGAG |
| R9 | 11197 | CTGGTGTAACTAAAGGATTAGCTGG |
| F10 | 11520 | TGTACATATTGCAAACGCATG |
| R10 | 12187 | GGTTCTTTACGTTCTGTTGCTCA |
| F11 | 11988 | GGAGTTCGATTAGTCTCAGCTAAAC |
| R11 | 12759 | ATCTGAGTTCAGACCGGCGTG |
| F12 | 12423 | GCATCACTAAAAGGCTGAGG |
| R12 | 13290 | CGCCTGTTTATCAAAAACAT |
| F13 | 13079 | AAGCTCTATAGGGTCTTCTCGTCC |
| R13 | 14013 | GTTCTGTGCAATTCAGGATGTCTTG |
| F14 | 13509 | GGTGGCTGGTTTAAAAGCCTACT |
| R14 | 14477 | GGTACTCATTCCATTCAGAGGAA |
| F15 | 14085 | CCAGTACATCTACTATGTTACGAC |
| R15 | 15047 | TGGTATAAGAGTACCATATCTAT |
| F16 | 14828 | GAAAGAGCGAACAAGTAAGCAAG |
| R16 | 110 | AAGGTGTATGATGCACGTTAGTT |

^a^ F: forward; R: reverse

**Table** **2** Mitochondrial genome sequences of insects in order Orthoptera used in this study, including GenBank accession numbers. The species sequenced for the first time in this study is highlighted in bold. Remaining sequences have been previously published [https://www.ncbi.nlm.nih.gov/]

| Order | Superfamily | Family | Genus | Species | Accession number | Dataset* |
| --- | --- | --- | --- | --- | --- | --- |
| Orthoptera | Acridoidea | Acrididae | Gastrimargus | *Gastrimargus marmoratus* | NC_011114 | 1 |
|  |  |  | Oedaleus | *Oedaleus decorus asiaticus* | NC_011115 | 1 |
|  |  |  | Locusta | *Locusta migratoria manilensis* | NC_014891 | 1 |
|  |  |  | Compsorhipis | *Compsorhipis davidiana* | NC_029408 | 1 |
|  |  |  | Angaracris | *Angaracris barabensis* | NC_025558 | 1 |
|  |  |  | Orinhippus | *Orinhippus tibetanus* | NC_023467 | 2 |
|  |  |  | Trilophidia | *Trilophidia annulata* | NC_027179 | 1 |
|  |  |  | Acrida | *Acrida cinerea* | NC_014887 | 1 |
|  |  |  | Pacris | *Pacris xizangensis* | NC_023919 | 2 |
|  |  |  | Ceracris | *Ceracris kiangsu* | NC_019994 | 1 |
|  |  |  | Ceracris | *Ceracris versicolor* | NC_025285 | 1 |
|  |  |  | Gonista | *Gonista bicolor* | NC_029205 | 1 |
|  |  |  | Schistocerca | *Schistocerca gregaria gregaria* | NC_013240 | 1 |
|  |  |  | **Chondracris** | ***Chondracris rosea*** | **NC_019993** | **1** |
|  |  |  | Xenocatantops | *Xenocatantops brachycerus* | NC_021609 | 1 |
|  |  |  | Peripolus | *Peripolus nepalensis* | NC_029135 | 0 |
|  |  |  | Qinlingacris | *Qinlingacris taibaiensis* | NC_027187 | 0 |
|  |  |  | Kingdonella | *Kingdonella bicollina* | NC_023920 | 2 |
|  |  |  | Prumna | *Prumna arctica* | NC_013835 | 0 |
|  |  |  | Yunnanacris | *Yunnanacris yunnaneus* | KX223964 | 0 |
|  |  |  | Yunnanacris | *Yunnanacris wenshanensis* | KX296781 | 0 |
|  |  |  | Curvipennis | *Curvipennis wixiensis* | KX553995 | 0 |
|  |  |  | Caryanda | *Caryanda sp.* | NC_030165 | 0 |
|  |  | Tristiridae | Tristira | *Tristira magellanica* | NC_020773 | 0 |
|  |  | Lithidiidae | Lithidiopsis | *Lithidiopsis carinatus* | NC_020775 | 0 |
|  |  | Lentulidae | Lentula | *Lentula callani* | NC_020774 | 0 |
|  |  | Pamphagidae | Humphaplotropis | *Humphaplotropis culaishanensis* | NC_023535 | 0 |
|  |  |  | Thrinchus | *Thrinchus schrenkii* | NC_014610 | 1 |
|  |  | Pyrgacrididae | Pyrgacris | *Pyrgacris descampsi* | NC_020776 | 0 |
|  | Pyrgomorphoidea | Pyrgomorphidae | Mekongiella | *Mekongiella xizangensis* | NC_014451 | 2 |
|  |  |  | Mekongiella | *Mekongiella kingdoni* | NC_023921 | 2 |
|  |  |  | Mekongiana | *Mekongiana xiangchengensis* | NC_014450 | 0 |
|  |  |  | Atractomorpha | *Atractomorpha sinensis* | NC_011824 | 1 |
|  | Eumastacoidea | Chorotypidae | Erianthus | *Erianthus versicolor* | NC_020045 | 3 |
|  | Tetrigoidea | Tetrigidae | Tetrix | *Tetrix japonica* | NC_018543 | 3 |

*: 0, 1, 2 and 3 represent flightless (non-Tibetan), flying, Tibetan grasshoppers and outgroup respectively.

**Table 3** Annotation of genes in the *Chondracris rosea* mitochondrial genome

| Gene | Strand^1^ | Nucleotide no. | Size(bp) | IN^2^ | Anticodon | Start codon | Stop codon |
| --- | --- | --- | --- | --- | --- | --- | --- |
| *tRNA^Ile^*  *tRNA^Gln^* | J  N | 1–65  68–136 | 65  69 | 0  2 | GAT  TTG | −  − | −  − |
| *tRNA^Met^* | J | 136–204 | 69 | −1 | CAT | − | − |
| *ND2*  *tRNA^Trp^*  *tRNA^Cys^* | J  J  N | 205–1227  1226–1292  1285–1351 | 1023  67  67 | 0  −2  −8 | −  TCA  GCA | ATG  − | TAA  − |
| *tRNA^Tyr^* | N | 1355–1423 | 69 | 3 | GTA | − | − |
| *COX1* | J | 1415–2959 | 1545 | −9 | − | ATC | TAA |
| *tRNA^Leu(UUR)^* | J | 2955–3020 | 66 | −5 | TAA | − | − |
| *COX2* | J | 3024–3707 | 684 | 3 | − | ATG | TAA |
| *tRNA^Asp^* | J | 3706–3773 | 68 | −2 | GTC | − | − |
| *tRNA^Lys^* | J | 3778–3848 | 71 | 4 | CTT | − | − |
| *ATP8* | J | 3873–4025 | 153 | 24 | − | ATA | TAA |
| *ATP6* | J | 4019–4708 | 690 | −7 | − | ATG | TAA |
| *COX3* | J | 4705–5493 | 789 | −4 | − | ATA | TAA |
| *tRNA^Gly^* | J | 5496–5563 | 68 | 2 | TCC | − | − |
| *ND3* | J | 5567–5917 | 351 | 3 | − | ATA | TAA |
| *tRNA^Ala^* | J | 5917–5982 | 66 | −1 | TGC | − | − |
| *tRNA^Arg^* | J | 5987–6053 | 67 | 4 | TCG | − | − |
| *tRNA^Asn^* | J | 6059–6124 | 66 | 5 | GTT | − | − |
| *tRNA^Ser(AGN)^* | J | 6124–6193 | 70 | −1 | GCT | − | − |
| *tRNA^Glu^* | J | 6195–6261 | 67 | 1 | TTC | − | − |
| *tRNA^Phe^* | N | 6260–6325 | 66 | −2 | GAA | − | − |
| *ND5* | N | 6309–8042 | 1734 | −17 | − | ATT | TAA |
| *tRNA^His^* | N | 8058–8125 | 68 | 15 | GTG | − | − |
| *ND4* | N | 8129–9463 | 1335 | 3 | − | ATG | TAA |
| *ND4L* | N | 9457–9750 | 294 | −7 | − | ATG | TAA |
| *tRNA^Thr^* | J | 9753–9821 | 69 | 2 | TGT | − | − |
| *tRNA^Pro^* | N | 9822–9886 | 65 | 0 | TGG | − | − |
| *ND6* | J | 9889–10410 | 522 | 2 | − | ATG | TAA |
| *CYTB* | J | 10419–11558 | 1140 | 8 | − | ATG | TAA |
| *tRNA^Ser(UCN)^* | J | 11564–11633 | 70 | 7 | TGA | − | − |
| *ND1* | N | 11650–12600 | 951 | 26 | − | ATA | TAG |
| *tRNA^Leu(CUN)^* | N | 12593–12660 | 68 | −8 | TAG | − | − |
| *16S* | N | 12661–13985 | 1325 | 0 | − | − | − |
| *tRNA^Val^* | N | 13986–14058 | 73 | 0 | TAC | − | − |
| *12S*  A+T-rich | N  J | 14059–14886  14887–15646 | 828  760 | 0  0 | −  − | −  − | −  − |

^1^ J indicates the majority strand; N indicates the minority strand.

^2^ a negative number indicates that the adjacent genes overlap; a positive number indicates an intergenic sequences

**Table 4** Results of selective pressure analysis (dN/dS ratios) of 13 PCGs in flying (F) vs. flightless (FL) lineages of grasshoppers based on 3 vs. 2 ratio model.

| Gene | 3-rate model dN/dS ratios | | | Is F > FL dN/dS? | Likelihood value | | P value |
| --- | --- | --- | --- | --- | --- | --- | --- |
|  | background | F | FL |  | 3-rate model | 2-rate model |  |
| *ATP6* | 0.0226 | 0.0241 | 0.0227 | TRUE | 6662.7191 | 6662.7610 | 0.7722 |
| *ATP8* | 0.0993 | 0.1184 | 0.1235 | FALSE | 2217.7157 | 2217.7224 | 0.9078 |
| *COX1* | 0.0129 | 0.0120 | 0.0118 | TRUE | 13456.5179 | 13456.5223 | 0.9258 |
| *COX2* | 0.0226 | 0.0248 | 0.0251 | FALSE | 6634.7861 | 6634.7886 | 0.9445 |
| *COX3* | 0.0311 | 0.0293 | 0.0296 | FALSE | 7859.9636 | 7859.9656 | 0.9501 |
| *CYTB* | 0.0216 | 0.0256 | 0.0240 | TRUE | 10976.1722 | 10976.2739 | 0.6519 |
| *ND1* | 0.0163 | 0.0142 | 0.0185 | FALSE | 8734.1161 | 8735.1165 | 0.1572 |
| *ND2* | 0.0741 | 0.0688 | 0.0899 | FALSE | 14318.0316 | 14320.1328 | 0.0404 |
| *ND3* | 0.0363 | 0.0423 | 0.0436 | FALSE | 3949.3734 | 3949.3803 | 0.9061 |
| *ND4* | 0.0356 | 0.0336 | 0.0258 | TRUE | 13258.8845 | 13260.7683 | 0.0523 |
| *ND4L* | 0.0137 | 0.0195 | 0.0151 | TRUE | 2552.0563 | 2552.2284 | 0.5574 |
| *ND5* | 0.0257 | 0.0206 | 0.0264 | FALSE | 17215.6676 | 17217.6036 | 0.0491 |
| *ND6* | 0.0267 | 0.0432 | 0.0612 | FALSE | 6576.7602 | 6577.7463 | 0.1602 |

**Table 5** Evidence of positive selection on the mitochondrial genes of flying and flightless grasshoppers based on site models.

| Gene | Model | −lnL | P level | M8 ω value | Positively selected sites |
| --- | --- | --- | --- | --- | --- |
| *ND2* | M8 | 13854.08 | 0.032 | 3.083 | 312 (0.980) |
|  | M8a | 13856.38 |  |  |  |

**Table 6** Genes and sites under positive selection of flying and flightless grasshoppers detected by Datamonkey.

| Gene | FEL (P<0.1) | FUBAR (pp>0.9) |
| --- | --- | --- |
| *ATP8* |  | 42 |
| *COX2* |  | 126 |
| *COX3* |  | 50,218 |
| *ND2* | 1,15,123,209 | 15,209,339 |
| *ND4* | 79,149 | 31,34,81 |
| *ND4L* | 15 | 15 |
| *ND5* | 374 |  |
| *ND6* | 43 | 101 |

**Table 7** Evidence of positive selection on the mitochondrial genes on each flying, flightless and common ancestral branches based on branch-site models. Branch letters correspond to those in Figure 1

| Gene | Branch | Model | -ln L | 2ΔlnL | P value | ω values | Positively selected site (BEB: P≥95%) |
| --- | --- | --- | --- | --- | --- | --- | --- |
| *ATP8* |  |  |  |  |  |  |  |
|  | FLD | MA | 2132.570 |  |  | ω0 =0.065 ω1 =1.000 ω2 = 484.437 | 26 L 0.990**  27 N 0.992** |
|  |  | MA0 | 2134.563 | 3.986 | 0.046 | ω0 =0.063 ω1 =1.000 ω2 =1.000 |  |
|  | FH | MA | 2129.466 |  |  | ω0 = 0.071 ω1 =1.000 ω2 = 551.179 | 12 L 0.957*  14 I 0.997**  27 N 0.998**  30 S 1.000**  32 K 0.968*  36 M 0.999**  47 K 0.972* |
|  |  | MA0 | 2133.868 | 8.805 | 0.003 | ω0 = 0.069 ω1 =1.000 ω2 =1.000 |  |
| *COX2* |  |  |  |  |  |  |  |
|  | A | MA | 6603.118 |  |  | ω0 = 0.022 ω1 =1.000 ω2 =189.076 | 32 L 0.970* |
|  |  | MA0 | 6605.324 | 4.413 | 0.036 | ω0 =0.022 ω1 =1.000 ω2 =1.000 |  |
| *COX3* |  |  |  |  |  |  |  |
|  | FF | MA | 7822.539 |  |  | ω0 = 0.028ω1 =1.000ω2 =998.972 | 257 S 0.994**  259 Y 0.971* |
|  |  | MA0 | 7826.690 | 8.302 | 0.004 | ω0 = 0.027ω1 =1.000ω2 =1.000 |  |
|  | FB | MA | 7822.514 |  |  | ω0 =0.028ω1 =1.000ω2 =178.862 | 6 S 0.999**  30 V 0.972* |
|  |  | MA0 | 7825.282 | 5.537 | 0.019 | ω0 =0.027ω1 =1.000ω2 =1.000 |  |
| *CYTB* |  |  |  |  |  |  |  |
|  | FB | MA | 10945.261 |  |  | ω0 =0.022ω1 =1.000ω2 =999.000 | 65 S 0.975* |
|  |  | MA0 | 10947.428 | 4.335 | 0.037 | ω0 0.022=ω1 =1.000ω2 =1.000 |  |
| *ND1* |  |  |  |  |  |  |  |
|  | FG | MA | 8696.440 |  |  | ω0 =0.015 ω1 =1.000 ω2 =15.122 | 278 R 0.993** |
|  |  | MA0 | 8699.307 | 5.733 | 0.017 | ω0 = 0.015 ω1 =1.000 ω2 =1.000 |  |
| *ND2* |  |  |  |  |  |  |  |
|  | FLB | MA | 14216.666 |  |  | ω0 = 0.076 ω1 =1.000 ω2 =20.599 | 99 S 0.976* |
|  |  | MA0 | 14218.891 | 4.450 | 0.035 | ω0 =0.077 ω1 =1.000 ω2 =1.000 |  |
|  | FLC | MA | 14205.501 |  |  | ω0 =0.075 ω1 =1.000 ω2 =470.242 | 23 S 0.993**  47 S 0.996**  144 I 0.997** |
|  |  | MA0 | 14210.232 | 9.462 | 0.002 | ω0 =0.075 ω1 =1.000 ω2 =1.000 |  |
|  | FB | MA | 14205.581 |  |  | ω0 =0.074 ω1 =1.000 ω2 =24.306 | 87 E 0.963*  89 E 0.954*  118 E 0.973*  211 V 0.989*  231 N 0.964*  242 L 0.999**  302 S 0.987* |
|  |  | MA0 | 14210.958 | 10.755 | 0.001 | ω0 =0.074 ω1 =1.000 ω2 =1.000 |  |
|  | FD | MA | 14212.679 |  |  | ω0 =0.076 ω1 =1.000 ω2 =27.870 | 47 S 0.964*  151 F 0.995** |
|  |  | MA0 | 14216.370 | 7.383 | 0.007 | ω0 =0.076 ω1 =1.000 ω2 =1.000 |  |
| *ND3* |  |  |  |  |  |  |  |
|  | FLB | MA | 3915.022 |  |  | ω0 = 0.041ω1 = 1.000 ω2 = 39.246 | 3 I 0.984*  97 V 0.989*  104 L 0.989*  105 Y 0.995**  107 E 0.986*  108 W 0.987*  112 A 0.997**  114 Q 0.986*  115 W 0.987*  116 A 0.995** |
|  |  | MA0 | 3918.780 | 7.517 | 0.006 | ω0 = 0. 040 ω1 = 1.000 ω2 = 1.000 |  |
| *ND4* |  |  |  |  |  |  |  |
|  | FE | MA | 13177.664 |  |  | ω0 =0.032ω1 =1.000 ω2 = 12.181 | 166 N 0.986* |
|  |  | MA0 | 13180.001 | 4.673 | 0.031 | ω0 =0.032ω1 =1.000 ω2 =1.000 |  |
|  | FLA | MA | 13174.763 |  |  | ω0 =0.032ω1 = 1.000ω2 = 9.460 | 368 S 0.992** |
|  |  | MA0 | 13177.812 | 6.098 | 0.014 | ω0 = 0.032ω1 =1.000 ω2 =1.000 |  |
| *ND4L* |  |  |  |  |  |  |  |
|  | FA | MA | 17067.474 |  |  | ω0 = 0.015 ω1 = 1.000 ω2 = 999.000 | 5 S 0.985* |
|  |  | MA0 | 17073.178 | 11.408 | 0.001 | ω0 = 0.0148 ω1 = 1.000 ω2 =1.000 |  |
| *ND5* |  |  |  |  |  |  |  |
|  | FA | MA | 2508.464 |  |  | ω0 =0.026 ω1 = 1.000 ω2 = 37.844 | 538 I 0.951* |
|  |  | MA0 | 2512.935 | 8.942 | 0.003 | ω0 =0.026 ω1 = 1.000 ω2 =1.000 |  |
| *ND6* |  |  |  |  |  |  |  |
|  | FC | MA | 6508.039 |  |  | ω0 = 0.056 ω1 =1.000 ω2 =999.000 | 138 N 1.000** |
|  |  | MA0 | 6513.310 | 10.544 | 0.001 | ω0 = 0.055 ω1 =1.000 ω2 =1.000 |  |

**Table 8** The feature and description of the positive selection sites detected in the mitochondrial PPGs of flying branches.

| **Genes**  **(***Locusta migratoria manilensis*) | **Positive selection sites** | **Feature key*** | **Description** |
| --- | --- | --- | --- |
| *ATP8* | 12 | Transmembrane | Helical |
|  | 14 | Transmembrane | Helical |
|  | 27 | Transmembrane | Helical |
|  | 30 |  |  |
|  | 32 |  |  |
|  | 36 |  |  |
|  | 42 |  |  |
|  | 47 |  |  |
| *COX3* | 6 | Domain |  |
|  | 30 | Domain |  |
|  | 50 | Transmembrane | Helical |
|  | 218 | Transmembrane | Helical |
|  | 257 | Transmembrane | Helical |
|  | 259 | Transmembrane | Helical |
| *ND2* | 1 | Signal peptide |  |
|  | 15 | Signal peptide |  |
|  | 47 | Domain | Proton_antipo_M |
|  | 87 | Domain | Proton_antipo_M |
|  | 89 | Domain | Proton_antipo_M |
|  | 118 | Domain | Proton_antipo_M |
|  | 123 | Domain | Proton_antipo_M |
|  | 151 | Transmembrane | Helical |
|  | 209 | Transmembrane | Helical |
|  | 211 | Transmembrane | Helical |
|  | 231 | Domain | Proton_antipo_M |
|  | 242 | Domain | Proton_antipo_M |
|  | 302 | Domain | NADH_dehy_S2_C |
|  | 339 | Transmembrane | Helical |
| *ND4* | 31 | Transmembrane | Helical |
|  | 34 | Transmembrane | Helical |
|  | 79 | Transmembrane | Helical |
|  | 81 | Transmembrane | Helical |
|  | 149 | Transmembrane | Helical |
|  | 166 | Domain | Proton_antipo_M |
| *ND4L* | 5 |  |  |
|  | 15 |  |  |
| *ND5* | 374 | Transmembrane | Helical |
|  | 538 | Transmembrane | Helical |
| *ND6* | 1 |  |  |
|  | 43 |  |  |
|  | 101 | Transmembrane | Helical |
|  | 138 |  |  |

* The functional information of genes identified as being under positive selection was derived from the uniprot (*Locusta migratoria manilensis*, <http://www.uniprot.org/>).

**Table 9** Results of selective pressure analysis (dN/dS ratios) of 13 PCGs in Tibetan (T) vs. non-Tibetan (NT) lineages of grasshoppers based on 3 vs. 2 ratio model.

| Gene | 3-rate model dN/dS ratios | | | Is T > NT dN/dS? | Likelihood value | | P value |
| --- | --- | --- | --- | --- | --- | --- | --- |
|  | background | T | NT |  | 3-rate model | 2-rate model |  |
| *ATP6* | 0.0194 | 0.0607 | 0.0306 | TRUE | 4889.3585 | 4891.9439 | 0.0230 |
| *ATP8* | 0.0797 | 0.0901 | 0.1954 | FALSE | 1554.1866 | 1555.1448 | 0.1662 |
| *COX1* | 0.0128 | 0.0190 | 0.0194 | FALSE | 9308.6069 | 9308.6106 | 0.9310 |
| *COX2* | 0.0291 | 0.0497 | 0.0290 | TRUE | 4779.8960 | 4781.8225 | 0.0497 |
| *COX3* | 0.0287 | 0.0520 | 0.0404 | TRUE | 5403.8502 | 5404.3376 | 0.3235 |
| *CYTB* | 0.0215 | 0.0318 | 0.0368 | FALSE | 7513.6313 | 7513.8286 | 0.5299 |
| *ND1* | 0.0164 | 0.0324 | 0.0287 | TRUE | 6070.3222 | 6070.4211 | 0.6565 |
| *ND2* | 0.0658 | 0.1284 | 0.0731 | TRUE | 9833.9800 | 9837.3332 | 0.0096 |
| *ND3* | 0.0400 | 0.0763 | 0.0448 | TRUE | 2780.7457 | 2781.6302 | 0.1835 |
| *ND4* | 0.0266 | 0.0534 | 0.0411 | TRUE | 9253.0949 | 9253.8852 | 0.2087 |
| *ND4L* | 0.0216 | 0.0312 | 0.0216 | TRUE | 1998.8511 | 1999.0796 | 0.4989 |
| *ND5* | 0.0247 | 0.0414 | 0.0375 | TRUE | 12468.1957 | 12468.3379 | 0.5938 |
| *ND6* | 0.0291 | 0.0650 | 0.0896 | FALSE | 4486.3637 | 4486.6907 | 0.4187 |

**Table 10** Genes and sites under positive selection of Tibetan and non-Tibetan grasshoppers detected by Datamonkey.

| Gene | FEL（P<0.1） | FUBAR （pp>0.9） |
| --- | --- | --- |
| *ATP6* | 52 | 52 |
| *COX2* | 185 |  |
| *COX3* | 50,75 | 50,75 |
| *ND2* | 15,147,332 | 15,51,147,332 |
| *ND3* |  | 102 |
| *ND4* | 17 |  |
| *ND5* | 8 |  |
| *ND6* | 122 | 89,122 |

**Table 11** Evidence of positive selection on the mitochondrial genes on each the Tibetan, non-Tibetan and common ancestral branches based on branch-site models. Branch letters correspond to those in Figure 2

| Gene | Branch | Model | -ln L | 2ΔlnL | P value | ω values | Positively selected site (BEB: P≥95%) |
| --- | --- | --- | --- | --- | --- | --- | --- |
| *ATP6* |  |  |  |  |  |  |  |
|  | Ta | MA | 4885.578 |  |  | ω0 =0.030 ω1 =1.000 ω2 = 684.433 | 44 N 0.959* |
|  |  | MA0 | 4887.721 | 4.285 | 0.038 | ω0 = 0.029 ω1 =1.000 ω2 =1.000 |  |
| *ATP8* |  |  |  |  |  |  |  |
|  | NTc | MA | 1500.698 |  |  | ω= 0.038 ω= 1.000 ω= 999.000 | 1 I 0.969*  11 S 0.967*  41 M 0.988* |
|  |  | MA0 | 1508.060 | 14.725 | 0.000 | ω= 0.036 ω=1.000 ω=1.000 |  |
| *ND1* |  |  |  |  |  |  |  |
|  | Ta | MA | 6015.771 |  |  | ω0 = 0.020 ω1 =1.000 ω2 =10.060 | 23 S 0.993** |
|  |  | MA0 | 6018.529 | 5.515 | 0.019 | ω0 =0.020 ω1 =1.000 ω2 =1.000 |  |
| *ND2* |  |  |  |  |  |  |  |
|  | NTc | MA | 9719.935 |  |  | ω0 =0.072 ω1 =1.000 ω2 =25.247 | 99 S 0.985*  316 K 0.967* |
|  |  | MA0 | 9723.485 | 7.100 | 0.008 | ω0 =0.071 ω1 = 1.000 ω2 = 1.000 |  |
|  | NTd | MA | 9710.311 |  |  | ω0 =0.070 ω1 =1.000 ω2 =309.485 | 23 S 0.990**  47 N 0.994**  144 I 0.996** |
|  |  | MA0 | 9715.063 | 9.506 | 0.002 | ω0 =0.069 ω1 = 1.000 ω2 = 1.000 |  |
|  | Tc | MA | 9715.955 |  |  | ω0 =0.070 ω1 =1.000 ω2 =82.363 | 160 I 0.965*  235 E 0.980*  279 A 1.000**  292 I 0.972*  304 N 0.965* |
|  |  | MA0 | 9718.776 | 5.641 | 0.018 | ω0 =0.070 ω1 =1.000 ω2 =1.000 |  |
|  | Td | MA | 9715.658 |  |  | ω0 = 0.069 ω1 =1.000 ω2 =15.463 | 3 S 0.992** |
|  |  | MA0 | 9718.164 | 5.012 | 0.025 | ω0 = 0.069 ω1 =1.000 ω2 =1.000 |  |
|  | B | MA | 9715.896 |  |  | ω0 = 0.070 ω1 =1.000 ω2 =34.738 | 47 N 0.978*  264 Q 0.999** |
|  |  | MA0 | 9720.061 | 8.330 | 0.004 | ω0 =0.070 ω1 =1.000 ω2 =1.000 |  |
| *ND3* |  |  |  |  |  |  |  |
|  | Tc | MA | 2761.478 |  |  | ω0 = 0.051ω1 = 1.000 ω2 =999.000 | 7 S 1.000**  18 V 0.970*  97 I 0.955* |
|  |  | MA0 | 2766.992 | 11.029 | 0.001 | ω0 = 0.052 ω1 = 1.000 ω2 = 1.000 |  |
| *ND4* |  |  |  |  |  |  |  |
|  | Td | MA | 9229.964 |  |  | ω0 = 0.035ω1 =1.000 ω2 =289.956 | 400 S 0.994** |
|  |  | MA0 | 9235.923 | 11.918 | 0.001 | ω0 = 0.035 ω1 =1.000 ω2 =1.000 |  |
|  | NTa | MA | 9230.812 |  |  | ω0 = 0.035ω1 =1.000 ω2 =11.673 | 368 G 0.992** |
|  |  | MA0 | 9234.114 | 6.603 | 0.010 | ω0 =0.035 ω1 =1.000 ω2 =1.000 |  |
| *ND5* |  |  |  |  |  |  |  |
|  | NTb | MA | 12328.729 |  |  | ω0 = 0.030 ω1 = 1.000 ω2 = 998.926 | 96 M 0.997**  104 N 0.965* |
|  |  | MA0 | 12334.406 | 11.355 | 0.001 | ω0 =0.030 ω1 =1.000 ω2 = 1.000 |  |
|  | Tb | MA | 12328.327 |  |  | ω0 =0.030 ω1 =1.000 ω2 = 998.908 | 319 D 0.976*  322 D 0.963* |
|  |  | MA0 | 12330.327 | 4.001 | 0.045 | ω0 = 0.030 ω1 =1.000 ω2 = 1.000 |  |
|  | Td | MA | 12325.203 |  |  | ω0 =0.029 ω1 =1.000 ω2 = 7.973 | 78 G 0.996**  298 T 0.992** |
|  |  | MA0 | 12327.328 | 4.250 | 0.039 | ω0 =0.029 ω1 =1.000 ω2 = 1.000 |  |
|  | A | MA | 12319.641 |  |  | ω0 =0.030 ω1 =1.000 ω2 =3.423 | 14 T 0.990**  175 F 0.950*  476 S 0.992**  511 K 0.998** |
|  |  | MA0 | 12321.924 | 4.565 | 0.033 | ω0 =0.030 ω1 = 1.000 ω2 =1.000 |  |

**Table 12** The feature and description of the positive selection sites detected in the mitochondrial PPGs of Tibetan grasshopper branches.

| **Genes**  (*Locusta migratoria manilensis* ) | **Positive selection sites** | **Feature key*** | **Description** |
| --- | --- | --- | --- |
| *ATP6* | 44 |  |  |
|  | 52 |  |  |
| *ND2* | 3 | Signal peptide |  |
|  | 15 | Signal peptide |  |
|  | 51 | Domain | Proton_antipo_M |
|  | 147 | Transmembrane | Helical |
|  | 160 | Transmembrane | Helical |
|  | 235 | Domain | Proton_antipo_M |
|  | 279 | Transmembrane | Helical |
|  | 292 | Transmembrane | Helical |
|  | 304 | Domain | NADH_dehy_S2_C |
|  | 332 | Transmembrane | Helical |
| *ND3* | 7 | Signal peptide |  |
|  | 18 | Signal peptide |  |
|  | 97 | Transmembrane | Helical |
|  | 102 | Transmembrane | Helical |
| *ND4* | 17 | Transmembrane | Helical |
|  | 400 |  |  |
| *ND5* | 8 | Transmembrane | Helical |
|  | 78 | Domain | Proton_antipo_N |
|  | 298 | Domain | Proton_antipo_N |
|  | 319 | Domain | Proton_antipo_N |
|  | 322 | Domain | Proton_antipo_N |

* The functional information of genes identified as being under positive selection was derived from the uniprot (*Locusta migratoria manilensis*, <http://www.uniprot.org/>).


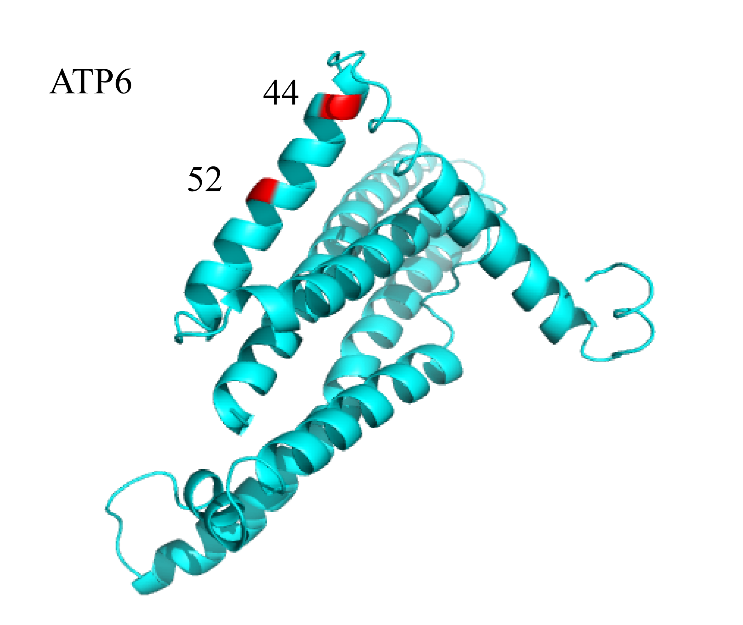

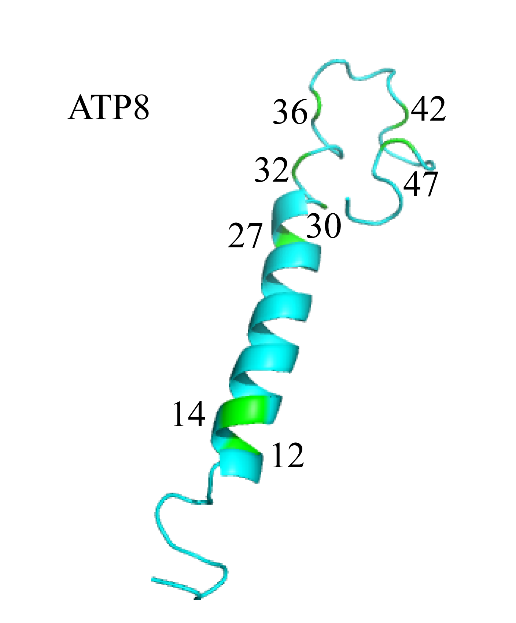

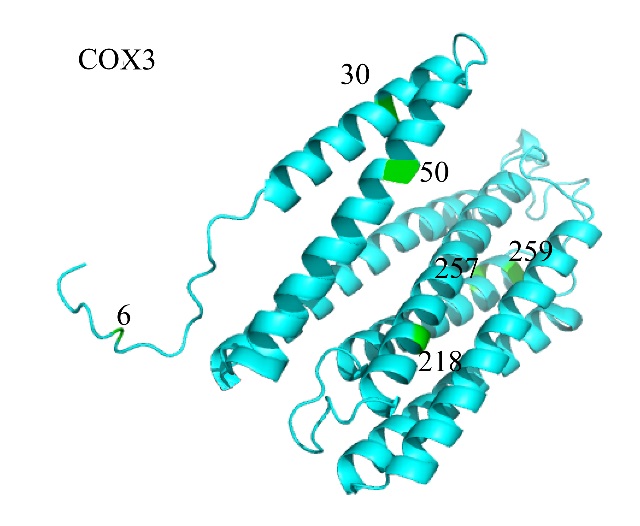


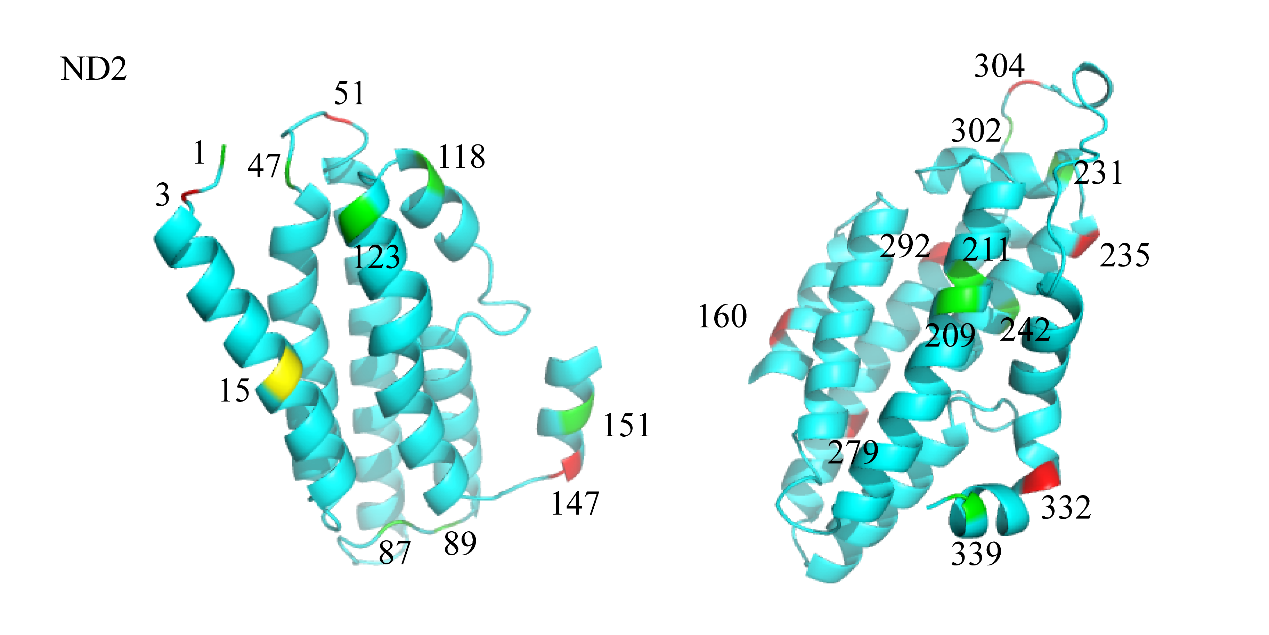


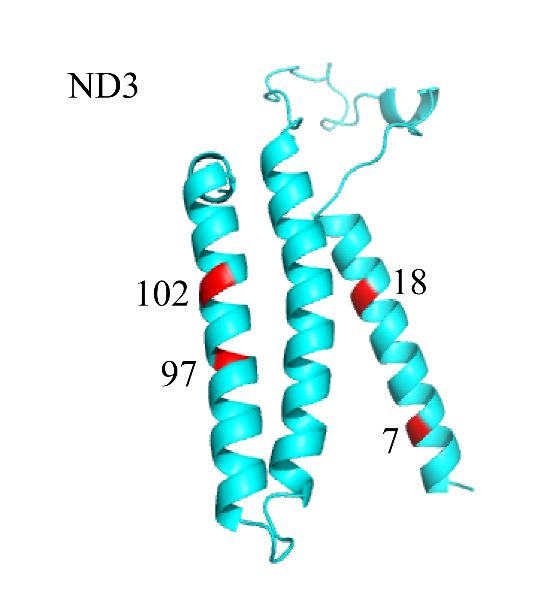

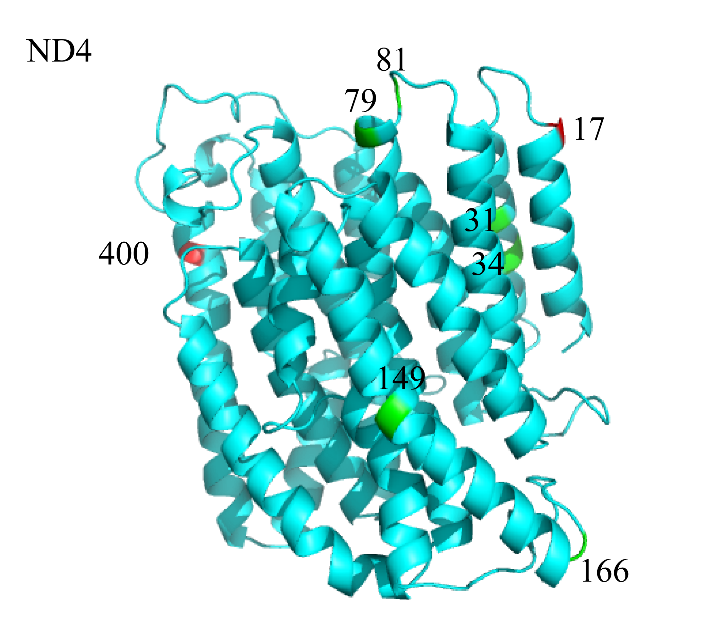


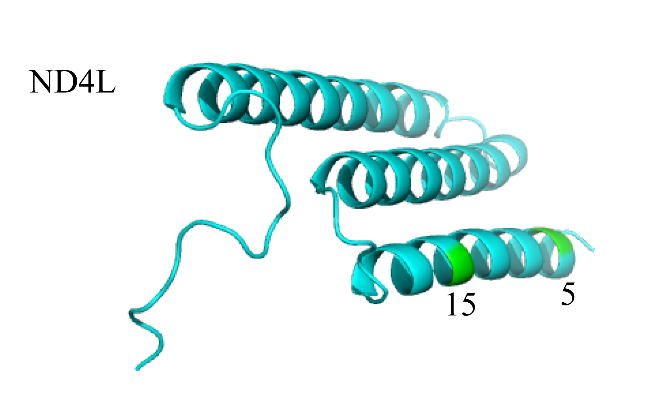

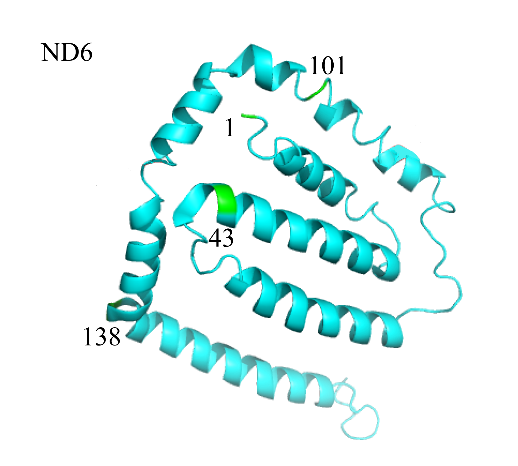

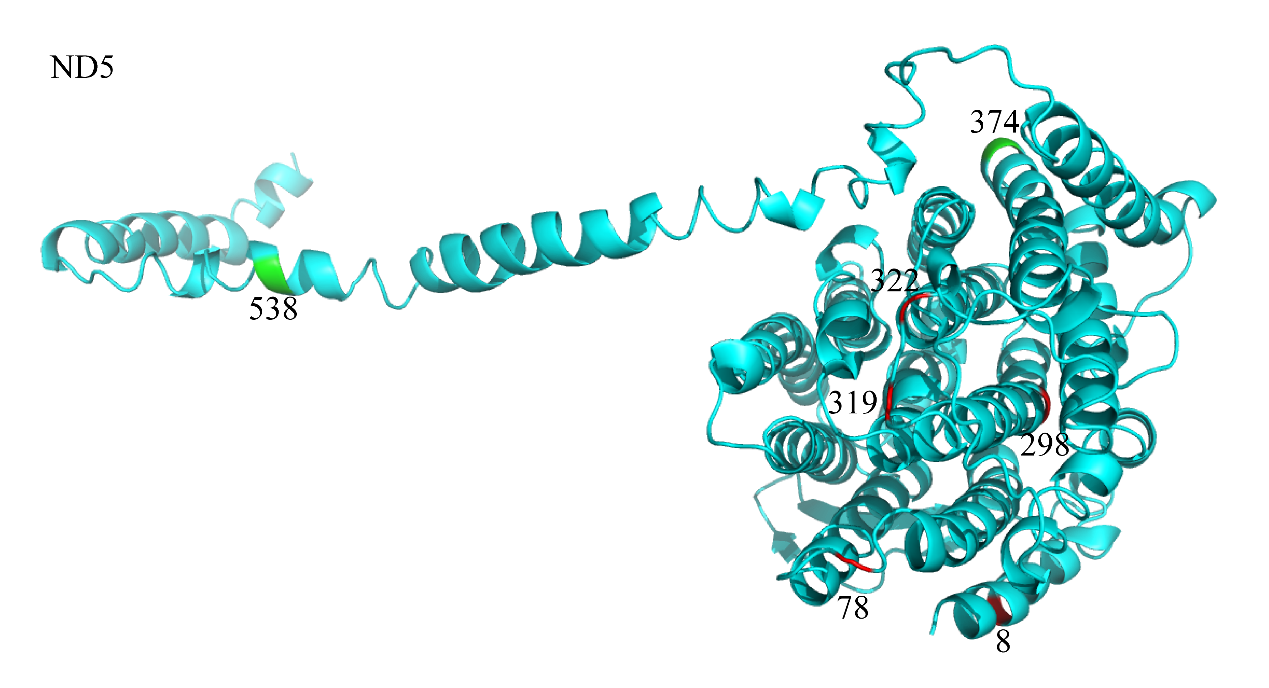


**FIGURE 3** Distribution of mutations in the three-dimensional structure of *ATP6*, *ATP8*, *COX3*, *ND2*, *ND3*, *ND4*, *ND4L*, *ND5* and *ND6* genes for the flying grasshopper and the Tibetan grasshopper. The green and red sites belong to the flying and Tibetan grasshoppers, separately. The yellow sites belong to the both.
